# Monthly Summary

**Published:** 1895-03

**Authors:** 


### Monthly Summary.

PULP-TREATMENT—CAP AND DRESSING.—A prominent feature in conservative treatment of the pulp is the means to protect it from pressure, and because of this necessity the means mostly used have given this mode of treatment the appellation of "Capping the pulp." The object of the cap is the avoidance of compression. Various means have been used to accomplish this. A common method has been to cover by a dressing the point of exposure; the bottom of the cavity being then lined with oxychloride of zinc or phosphate of zinc, a coating of gutta-percha varnish being interposed between the dressing and the protective material. As this means is complicated, somewhat uncertain, not applicable in shallow cases, and in those very difficult of access, I have preferred to depend upon a metal cap as being simpler and better under control than the above described method.

This kind of cap not only insures to prevent compression, but serves to contain the dressing, which completely occludes the space between the cap and the pulp. These caps may be made of thin platinum or of gold, the former being preferable. In form they should be circular to meet the generality of cases, and oval to accommodate broad exposures. They are most easily made by punching them out with the round and oval leather puncher of the hardware shops upon the end of a block of hard wood. This action gives them sufficient concavity for the purpose. For ordinary purposes they should be quite thin, but when it is considered safe to use gold fillings the cap should be sufficiently thick to resist the force of the packing.

It is necessary that the space under the cap should be completely closed, otherwise it would fill with fluid effused from the pulp, which quickly would undergo putrefactive changes resulting in the evolution of gas and consequently of compression of the organ beneath. In the earliest attempts at capping the pulp this became the condition, the only thought then being to protect from the pressure of the instruments. It was then generally supposed that the pulp underwent projection into the space and became strangulated, but all the indications now point to compression by gases being the cause of the almost immediate disturbance.

The dressing I have found most acceptible is formed by rubbing up oxid of zinc into a paste with carbolic acid and oil of cloves of equal measure. The consistency of this should be such that, while being so plastic that the paste will flow out around the cap when it is gently pressed in position, it will not be so thin as to flow out of the cap when it is held on its edge.

The ingrediency of the dressing is based upon these considerations: The menstrum is antiseptic, and possesess some anæsthetic value. It also remains unchanged within the space, and in time becomes, from the dissipation of the menstrum, somewhat firm in its character. The therapeutic action of the menstrum is mild, and is employed for the reason that it is slowly given up by the oxid of zinc, and therefore, makes an acceptible dressing.

I would call your attention to the value of the combination of hypophosphite of lime with zinc oxid, as, with the previously stated menstrum. This I have used in many of my earlier experiments, and have lately returned to it in cases of a less promising character than those considered within the lines of easy treatment.—Jack (L.), *International.*

---

TO REMOVE PLASTER IMPRESSION FROM THE TRAY WHEN IT HANGS.—Place a Bunsen burner on a folded towel; pass the tray (cup down) back and forth over the flame. Steam will be generated between plaster and tray, and it will pass loose and fall on the folded towel.—*The Busy Dentist.*

SENSITIVE DENTINE.—Moisten a pledget of cotton with alcohol, take up crystals of nitrat of silver and place in contact with the sensitive dentine, retaining in place by molding over it a gutta-percha cup. Leave in place for a day or two, when the dentine can be excavated without pain.—S. W. Foster in *Items of Interest.*

---

DIAGNOSIS IN OBSCURE CASES.—A small pledget of cotton wet with ether, and placed near the neck of each tooth separately, will promptly show which have living pulps and which have not. The rapid evaporation of the ether produces intense cold—agreeable to the inflamed part or where there is a dead pulp, but painful where there is a living pulp.—H. E. BEACH in *Items of Interest.*

---

CAST SOLID GOLD CUSPS.—Place a small piece of moldine—best a little dry and stiff—on the soldering block; make the surface flat and coat with soapstone. Into this press the cusps of a suitable tooth, On the die thus made press gold scraps, fillings, etc., and fuse by blow-pipe. While in the molten state press into the die with a flat-end carbon stick. Solid gold dummies may be cast in the same way.—*Items of Interest.*

---

PULP CAPPING IN DECIDUOUS TEETH.—Rotate a round-faced burnisher with pressure, on a piece of thin platina resting against soft wood, till a little saucer-like depression is formed. Cut out this little cup, and fill it with a paste made of oxid of zinc, carbolic acid and oil of cloves, equal parts, Invest this carefully over the exposed pulp, and fill the cavity with oxiphosphate.—*Items of Interest.*

